# Atypical Chemokine Receptor 1 Polymorphism can not Affect Susceptibility to Hepatitis C Virus

**DOI:** 10.4274/balkanmedj.2016.0766

**Published:** 2017-08-04

**Authors:** Shu-Ting Zhang, Ming Shi, Lin-Nan Shao, Shi-Hang Zhou, Wei-Jian Yu, Mei Chen, Nan Xiao, Ying Duan, Ling-Zi Pan, Ni Wang, Wen-Qian Song, Yue-Xin Xia, Li Zhang, Ning Qi, Ming Liu

**Affiliations:** 1 Dalian Blood Center, Liaoning, China; 2 Dalian University School of Medicine, Liaoning, China; 3 Department of Cell Biology, Dalian Medical University, Liaoning, China

**Keywords:** Atypical chemokine receptor 1, polymorphism, Hepatitis C virus, susceptibility

## Abstract

**Background:**

Hepatitis C virus has infected 130 to 150 million individuals globally. Atypical chemokine receptor 1 has become a focus of research because of its diverse roles in different diseases. However, little is known regarding the association of atypical chemokine receptor 1 polymorphism with susceptibility to hepatitis C virus.

**Aims:**

To determine the association of an atypical chemokine receptor 1 polymorphism (rs12075) with hepatitis C virus susceptibility.

**Study Design:**

Case-control study.

**Methods:**

We collected blood samples from 231 patients infected with hepatitis C virus and 239 blood donors as control subjects. Genotyping of atypical chemokine receptor 1 was performed using a 5ˊ-nuclease assay with TaqMan-minor groove binding probes. Comparisons between hepatitis C virus-infected patients and control subjects were assessed using Fisher’s exact test.

**Results:**

The genotype frequencies of FY*A/FY*A, FY*A/FY*B and FY*B/FY*B were 86.1%, 13.9% and 0% in the patient group, and 86.2%, 13.4% and 0.4% in the control group, respectively. The difference in atypical chemokine receptor 1 genotype frequencies between hepatitis C virus-infected patients and control group was not significant (p=1.00, OR=1.004, 95% CI=0.594-1.695). FY*A and FY*B allele frequencies were 93.1% and 6.9% in the patient group, and 92.9% and 7.1% in the control group, respectively. The difference in atypical chemokine receptor 1 allele frequencies between hepatitis C virus-infected patients and the control group was not significant (p=1.00, OR=0.972, 95% CI=0.589-1.603).

**Conclusion:**

Our result indicates that atypical chemokine receptor 1 polymorphism (rs12075) does not affect susceptibility to hepatitis C virus.

A global public health problem in both developing and developed countries, hepatitis C virus (HCV) has infected 130 to 150 million individuals globally according to the World Health Organisation. The annual increase of HCV infection is approximately 3.5 million (1). A significant number of those who are chronically infected will develop liver cirrhosis, hepatocellular cancer and liver failure (2), and more than 70% of patients with HCV infection develop chronic infections leading to end-stage liver diseases or even death (3). Only 15-45% of infected patients successfully eliminate the virus spontaneously. In Asia, more than 100 million individuals may be chronically infected with HCV (4). Recent data suggest that the prevalence of HCV infection ranges from 0.43% to 3.2% in the Chinese population, and varies geographically and temporally in China (5). HCV is a blood borne virus and the major modes of transmission are through inadequate sterilisation of medical equipment, unsafe injection practices, sexual contact and unscreened blood or blood product transfusion.

The Duffy blood group antigens, recently renamed atypical chemokine receptor 1 (ACKR1) (6), are glycoproteins expressed mainly in erythrocytes, in endothelial cells throughout the body, and in cerebellar neurons (7). The Duffy gene is situated on chromosome 1 at the q22-q23 position. Three main alleles have been characterised: FY*A (encoding Fy^a^), FY*B (encoding Fy^b^), and FY*B^ES^ (8). FY*A and FY*B are differentiated by a single nucleotide polymorphism (SNP), rs12075 (9). FY*B^ES^ corresponding to the Fy (a-b-) phenotype and a lack of ACKR1 in erythrocytes, is disrupted by a SNP, rs2814778 (T-33C) in the GATA box motif of the gene’s promoter region (10). The phenotype of Fy (a-b-) is present in most West Africans (>95%) and in approximately 68% of African American individuals, but is rare in the Chinese population (11).

ACKR1 is responsible for interactions with chemokines (12). Moreover, ACKR1 has also been described as a chemokine “sink” to bind and clear chemokines from sites of overproduction (13). There is abundant evidence to suggest that ACKR1 polymorphisms are associated with some diseases, such as chronic periodontitis, breast cancer and Plasmodium vivax malaria (14,15,16). Several studies have demonstrated that the erythrocyte complement receptor is associated with HCV (17,18,19). Moreover, as another receptor on erythrocytes, ACKR1 polymorphism may play a critical role in human immunodeficiency virus (HIV) infection (20,21). However, little is known regarding the association of ACKR1 polymorphism with susceptibility to HCV.

In this study, we investigate the association of ACKR1 polymorphism (rs12075) with susceptibility to HCV. If rs12075 is a risk factor, exploring this specific indicator in patients with HCV infection could benefit the development of novel strategies to detect and prevent infection at the early stage.

## MATERIALS AND METHODS

### Patients and samples

A total of 231 HCV-infected patients who were in treatment in Dalian infectious hospital were recruited to participate in this study, and 239 unrelated healthy blood donors were recruited as a control group. To avoid sampling error and inter-observer variation, and to avoid introducing bias, patients with HCV infection were recruited according to the following criteria: presence of HCV antibody and HCV RNA with abnormal liver function tests and/or biopsy evidence of HCV related liver diseases. Ethics committee approval was received for this study from the local ethics committee. Written informed consent was obtained from all participants in the study.

The whole peripheral blood specimens were collected in vacuum tubes with EDTA as anticoagulant. DNA was extracted from peripheral lymphocytes using a DNA isolation kit (RBCBioscience, Taipei, Taiwan) following the manufacturer’s instructions.

### ACKR1 genotyping

ACKR1 was genotyped using a 5’-nuclease assay (NA) with TaqMan minor groove binding (MGB) probes. The primers and probes were synthesised by Applied Biosystems (Table 1). The polymerase chain reaction (PCR) mixtures included 1 μL of purified genomic DNA, 10 μL of 2x TaqMan Universal PCR Master Mix (Applied Biosystems, Foster City, CA, USA), 0.9 μL of each primer (20 μM), 0.2 μL of each probe (20 μM) and 6.8 μL of distilled water in a final reaction volume of 20 μL. The 5’-NA was performed on an ABI Prism 7300 sequence detection system (Applied Biosystems) using a cycle of 95 °C for 10 min, followed by 40 cycles at 95 °C for 15 s and 60 °C for 1 min. The results were sorted into three distinct groups, corresponding to the three genotypes, homozygous FY*A/FY*A, FY*B/FY*B and heterozygous FY*A/FY*B.

### Statistical analysis

Fisher’s exact tests and Mann-Whitney U-tests were used for comparisons based on gender and age, respectively. Allele frequencies and genotype frequencies were obtained by the direct counting method. To evaluate the distribution of gene frequencies in two groups, the classical test for Hardy-Weinberg equilibrium (HWE) was examined. Comparisons between HCV-infected patients and the control group were assessed using Fisher’s exact test. P values <0.05 were considered statistically significant. The strength of the relationship was estimated by calculating the odds ratios (OR) and 95% confidence intervals (CI).

## RESULTS

**Figure 1:**
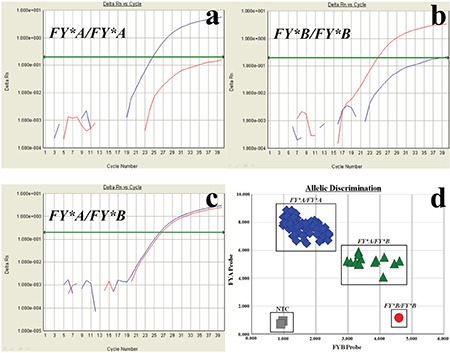
Representative amplification curves of homozygous FY*A/FY*A (a), homozygous FY*B/FY*B (b) and heterozygous FY*A/FY*B (c). End-point fluorescent signals from several samples (d) Homozygous FY*A/FY*A showed an increased fluorescence along the Y-axis, homozygous FY*B/FY*B along the X-axis, whereas heterozygous FY*A/FY*B showed an increase in fluorescence intensity along both the X-axis and the Y-axis. Blue curves: fluorescent intensity of dye FAM. Red curves: fluorescent intensity of dye VIC. NTC: No Template Control.

Overall, 231 patients with HCV infection and 239 healthy individuals were genotyped by using the TaqMan-MGB probes. Representative analysis results are presented in Figure 1. No significant deviation was found from HWE within each group (p=0.26 in HCV-infected patients; p=0.84 in the control group). Table 2 summarises the ACKR1 genotype and allele frequencies in the patient and control groups. The genotype frequencies of FY*A/FY*A, FY*A/FY*B and FY*B/FY*B were 86.1%, 13.9% and 0% in the patient group, and 86.2%, 13.4% and 0.4% in the control group, respectively. The difference in ACKR1 genotype frequencies between HCV-infected patients and the control group was not significant (p=1.00, OR=1.004, 95% CI=0.594-1.695). Moreover, FY*A and FY*B allele frequencies were 93.1% and 6.9% in the patient group, and 92.9% and 7.1% in the control group, respectively. The difference in ACKR1 allele frequencies between HCV-infected patients and the control group was not significant (p=1.00, OR=0.972, 95% CI=0.589-1.603).

## DISCUSSION

ACKR1 was identified as a blood group antigen that was expressed on the human red blood cell surface in 1950 (22). In the early 1990s, ACKR1 was found to be a receptor for interleukin 8 and other inflammatory chemokines (13,23). In recent years, ACKR1 SNPs have become the focus because of their complex and critical roles in diseases. Yang et al. (16) suggested that ACKR1 polymorphism could affect metastasis of breast cancer and the FY*B allele could decrease the possibility of lymph node metastasis. He et al. (20) found that ACKR1 polymorphism was associated with a 40% increase in the odds of acquiring HIV-1. King et al. (14) demonstrated that ACKR1 polymorphism affected binding of Plasmodium vivax Duffy binding protein to erythrocytes and FY*A was associated with a lower risk of clinical Plasmodium vivax in humans compared with FY*B.

In the present study, we detected the polymorphism (rs12075) of ACKR1 in 231 patients with HCV infection and 239 healthy individuals. The results showed in both the HCV-infected patients and in the control group, the FY*A allele and FY*A/FY*A genotype were absolutely predominant, while FY*B/FY*B was rare. These results were in good accordance with the distribution of the allelic groups found around the world (11). Our study was based on the hypothesis that ACKR1 polymorphism, which can affect HIV susceptibility, might also be associated with HCV infection. We here tested the hypothesis that this genetic variant (rs12075) in ACKR1 is a risk factor for HCV infection. However, our results showed that there was no significant correlation (p>0.05) with the allele and genotype frequency distribution for the ACKR1 between HCV-infected patients and the control group. Lettow et al. (24) also observed a similar result in Caucasians - the allele and genotype frequency distribution for ACKR1 (rs12075) did not differ between the control group and HCV-infected patients. Thus, we demonstrate that ACKR1 polymorphism (rs12075) does not affect susceptibility to HCV. It is probable that ACKR1 on erythrocyte membranes cannot interact with HCV, so ACKR1 polymorphism is not associated with HCV susceptibility.

ACKR1 represents an atypical chemokine receptor that can bind promiscuously to various chemokines belonging to the CC and CXC families (25). In HCV infection, the most prominent chemokines are the CC and CXC families, and they are highly expressed (26). Studies have shown that the CC and CXC families are associated with liver injury (27). Moreover, the CC and CXC families can recruit various leukocyte subsets into the liver during the progression of HCV infection (28). Recent studies have found that ACKR1 polymorphism is associated with serum levels of CCL2 belonging to the CC family (29,30). Based on the above findings, in further studies, we will focus on whether there is a correlation between ACKR1 polymorphism and liver fibrosis progression associated with HCV infection.

In conclusion, our study indicates that ACKR1 polymorphism (rs12075) does not affect susceptibility to HCV and is not a risk factor for HCV infection.

## Figures and Tables

**Table 1:**
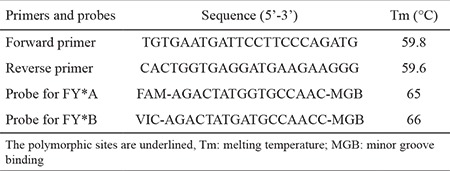
Primers and TaqMan-MGB probe sequences

**Table 2:**
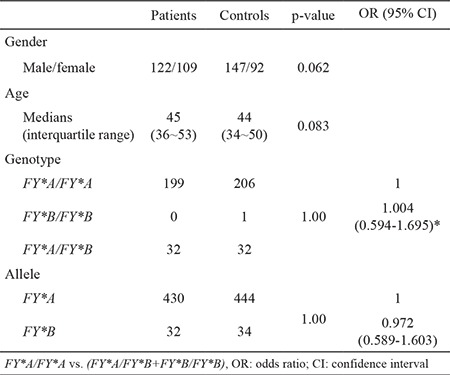
The distributions of the ACKR1 genotypes and alleles in hepatitis C virus-infected patients (n=231) and the control group (n=239)
